# A novel on high voltage gain boost converter with cuckoo search optimization based MPPTController for solar PV system

**DOI:** 10.1038/s41598-024-58820-2

**Published:** 2024-04-12

**Authors:** T. Mariprasath, C. H. Hussaian Basha, Baseem Khan, Ahmed Ali

**Affiliations:** 1 K.S.R.M College of Engineering (Autonomous), Kadapa, India; 2 Electric Vehicle R&D Lab, NITTE Meenakshi Institute of Technology, Bangalore, India; 3 Department of Electrical and Computer Engineering, Hawassa University, Hawassa 05, Ethiopia; 4 Department of Electrical and Electronic Engineering Technology, University of Johannesburg, Johannesburg, South Africa

**Keywords:** Solar PV, Boost converter, Grey wolf optimization, Cuckoo search optimization, Flower pollination, Particle swarm optimization, Maximum power point tracking (MPPT) technique, Partial shading, Engineering, Electrical and electronic engineering, Energy infrastructure

## Abstract

Traditionally, isolated and non-isolated boost converters are used for solar photovoltaic systems (SPV). These converters have limitations such as low voltage gain, less voltage ripples, temperature dependence, high voltage stress across the switches, and being bulky in size. Besides, the solar PV system also has non-linear characteristics between I–V and P–V, and the energy yield potential is affected by partial shading phenomena. Therefore, maximum power point tracking (MPPT) is being added to the SPV system to get the maximum output power under steady and dynamic climate conditions. Although the conventional MPPT has drawbacks such as less accuracy in predicting the MPP under partial shading conditions, low tracking speed, and more ripples, Hence, the research proposes a stackable single switch boost converter (SSBC) with a Cuckoo search MPPT controller for the SPV system. The efficiency of the proposed circuit topology has been compared with conventional boost converters with various MPPTs. Subsequently, the accuracy of tracking true MPPT by CSO is compared with that of PSO and FPNA. The results show, that the CMPPT with CBC has produced more ripples, whereas the BMPPT with SSBC produces ripple-free power under steady conditions. It is also observed that SSBC with BMPPT produces more power than SSBC with TMPPT. The efficiency of SSBC with BMPPT is better than other combinations. Finally, a prototype model has been developed and verified.

## Introduction

**Figure 1:**
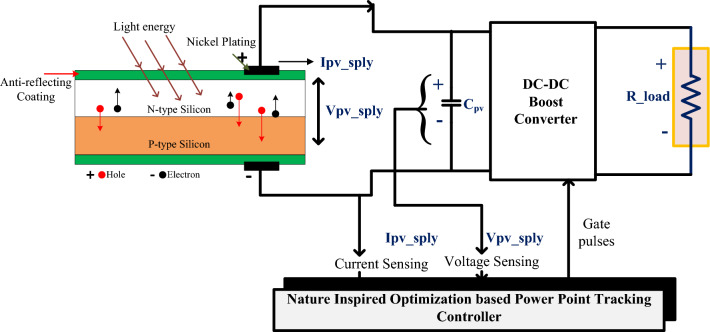
Principle operation of SPV cell.

Due to urbanization, industrialization, and long-term changes in people's social and economic status, the need for electricity has grown quickly around the world. Because of this, the power industry is adding to its ability to make power through a program called capacity addition ^1–5^. The growth rate of power generation is shown in Fig. 1. Traditional power plants use fossil fuels such as coal, petrol, and diesel as a primary resource for generating electrical energy ^6^. India's government says that 60% of the country's electricity comes from thermal power plants. It includes 52.6% from coal-based power plants, 1.7% from lignite, 6.5% from gas, and 0.1% from diesel, where the remaining 40 percent were obtained from Renewable Energy Resources (RER), respectively. Conventional power plants have problems like not having enough resources, fuel prices that change often, and putting harmful gases into the air. So, RER is playing a significant role in electrical energy generation ^7–9^.

RER is solar, wind, hydro, tidal, etc. ^10^. By using suitable conversion technology, electrical energy is extracted from RER. For example, wind power plants convert kinetic energy into electrical energy by using blades. Whenever air interacts with blades, it results in rotation, which means that it generates kinetic energy. This kinetic energy is used to rotate the turbine. It is connected to the wind generator, so it uses the induction principle to make electricity. The method has some problems, such as a high initial cost, hard maintenance, and a need for more land. In addition, it creates power quality issues on the grid ^11–15^. So, the compensating equipment, which costs more, is needed to make sure that the power quality is right. However, India occupies the fourth place for harnessing electrical energy from wind power plants. The total installed capacity in India is 38.789 GW ^16^.Solar power plants are also growing at a slow rate because the cost of the SPV cell is going down slowly but surely every day. PV power generation is based on the photovoltaic effect. SPV is made of P-type semiconducting materials sandwiching N-type semiconductor materials. P-type semiconductor materials have fewer electrons than N-type semiconductor materials. When the sun shines on the solar cell, the electrons in the N-type material become energized, causing the N layer to move toward the player, as shown in Fig. 1. The current created by the photovoltaic effect is collected by connecting a load across the P-type and N-type semiconductor materials ^17^.

A single PV cell can generate up to 0.7 V, which is not enough to drive the load ^18^. As a result, the number of PV cells is connected in series to form the solar cell strings. Then, the number of cell strings is connected to form the module. The number of modules is connected to form the string. This process significantly increases the incident area of solar irradiation, known as the catchment area, so that the power generation capacity of SPV is increased ^19^. There are currently three types of SPV cell technologies in use: mono, poly, and thin film. Among them, monocrystalline is more costly than others. Monocrystalline, on the other hand, has efficiency comparable to all others. Furthermore, monocrystalline PV requires less space than others to generate a specific wattage. But cost-wise, polycrystalline is preferable to others ^20–25^.The SPV energy yield potential depends a lot on the amount of light, the temperature of the cell, and the weather. The SPV was unable to produce constant power the whole day. Because the amount of available irradiation in the Earth's atmosphere varies over time, the relation between the voltage and current is non-linear, so extracting maximum power from SPV is a crucial task.

Hence, a new technology called MPPT is incorporated with the SPV system for extracting maximum power. The role of MPPT is to track the maximum power point and keep the SPV working on that particular point. Meanwhile, the MPP point also changes with time; therefore, MPPT continuously tracks the MPP based on the present and past measurements ^26^. All MPPT techniques track the MPP by using present and previous power quantity measurements. The methodology to be used has been a key parameter for tracking the MPP ^27–29^.Moreover, conventional MPPT can track the MPP under steady climatic conditions. When the climate changes quickly, conventional MPPT can't find the MPP because there are so many peaks at the same time. Therefore, accurate MPPT techniques are required to track the MPP. MPPT is the subject of numerous studies. Traditional boost converters have low voltage gain and high voltage stress across switches. Also, the problem of diode reverse recovery voltage is made worse by the fact that a high-duty cycle is needed to get a high voltage gain ^30^.

Isolated and non-isolated converters are the two broad categories of converters ^31^. Isolated boost converters achieve high voltage by varying the turn ratio of the transformers. Isolated DC-DC converters have limitations such as low efficiency, low immunity to electromagnetic interference, a larger size, and a higher peak voltage that can damage other electronic components used in the converter ^32–35^. While non-isolated converters are smaller in size, have a simpler structure, and are less expensive, the voltage gain ratio is very low. It can be overcome by interleaved boost converters.Nevertheless, it produced current ripples and high voltage stress across the switches. So, cascade converters are made to improve the ratio of voltage conversion, but they need more power electronics parts, which makes the circuit more complicated and increases current ripples. ^36^. So, the research work suggests a DC-to-DC boost converter with a single switch that can be stacked to improve the voltage level of the boost converter. The cuckoo search MPPT is used to change the duty cycle of the proposed boost converter. Then, the results are compared to those of traditional boost converters with traditional MPPT controllers. Also, a comparison was made between CS MPPT with SCB and SCB with bio-inspired MPPT controllers.

## Advanced adaptive Cuckoo search algorithm

The Cuckoo Search Algorithm is a meta-heuristic algorithm. The Cuckoo search algorithm was inspired by the fact that some species lay their eggs in the nests of other birds for increased survival ^37^. Let's say that the host bird finds the egg of the other bird in its net. Both the egg and the net would be destroyed, and new nets would be built in other places. From a mathematical point of view, each egg in the net is called a solution ^38^. The probability of the survival of a cuckoo bird's egg is possible when its appearance is similar to that of the host bird. Perhaps the worst solution is when the cuckoo bird egg is identified by the host bird ^39^. The algorithm is developed by the following three key conditions: At first, a cuckoo lays one egg, which is randomly placed in the host bird's nest. The eggs are called solution, and they are stored in the host nest. Moreover, an egg in the nest is called a set of solutions, whereas a cuckoo egg is called a new solution. The next generation is possible with a high-quality egg and the best nest. This high-quality egg is referred to as the best solution because it is close to the best value ^40^. It can replace the less-fitting solutions. Finally, the number of hosts is not variable. Mathematically, the population is defined as the number of host nests, whereas the number of host birds that find the egg is defined as the worst solution ^41–44^.

The cuckoo bird is used to test the flight method to find the best solution or a new solution that is better than the previous one ^45^. There are three ways to find the best solution for the levy flight: walking, running, or a random walk. The first term represents moving at a regular pace by lifting and setting down each foot in turn and never having both feet at ground level. The run represents a faster speed than the walk. Random walking is a mix of step-by-step walking in different directions. Whereby the cuckoo search algorithm is based on the random walk search process, respectively. At first, the cuckoo bird uses a pattern called smaller steps followed by larger steps to figure out where the best place is to lay an egg ^46^. The cuckoo bird is finding a suitable net, as described by the Levy flight. According to the power law, the step size of Levy's flight is estimated because Levy's flight is used in a random walk to find the nest (refer to Eq. (1)).

1 $$ {\text{y}} = {\text{l}}^{{ - {1}}} $$

**Figure 2:**
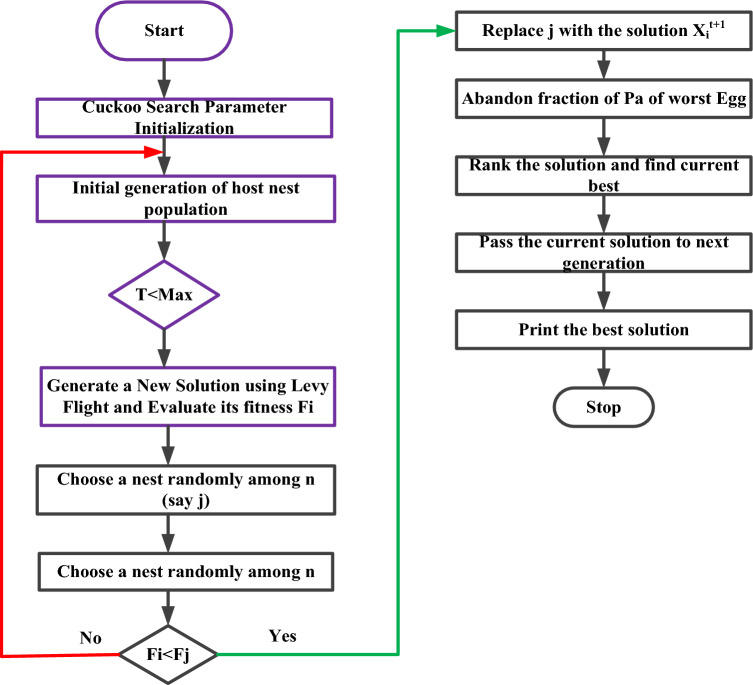
Working flow of the cuckoo search algorithm.

The flow chart of the Cuckoo Search Algorithm is depicted in Fig. 2. First, you need to set up the parameters, like the number of host nests (n), the chance that the host bird will find the cuckoo egg (Pa), and the maximum number of iterations (Max). Subsequently, check whether the number of iterations is less than the maximum number ^47^. If the condition is true, the levied flight search method is used to come up with a new solution, and the fitness is then checked. Hence, a new nest is searched randomly for the next solution. Once again, the fitness of the host egg is compared to the fitness of the cuckoo egg; assume that the cuckoo egg is healthier than the host egg and that the cuckoo egg replaces the host egg. Whereas the host egg fitness value is higher, the cuckoo egg solution is the worst. So, the cuckoo egg is thrown away from the nest, which generates a new solution by using Levy flight ^48^.

**Figure 3:**
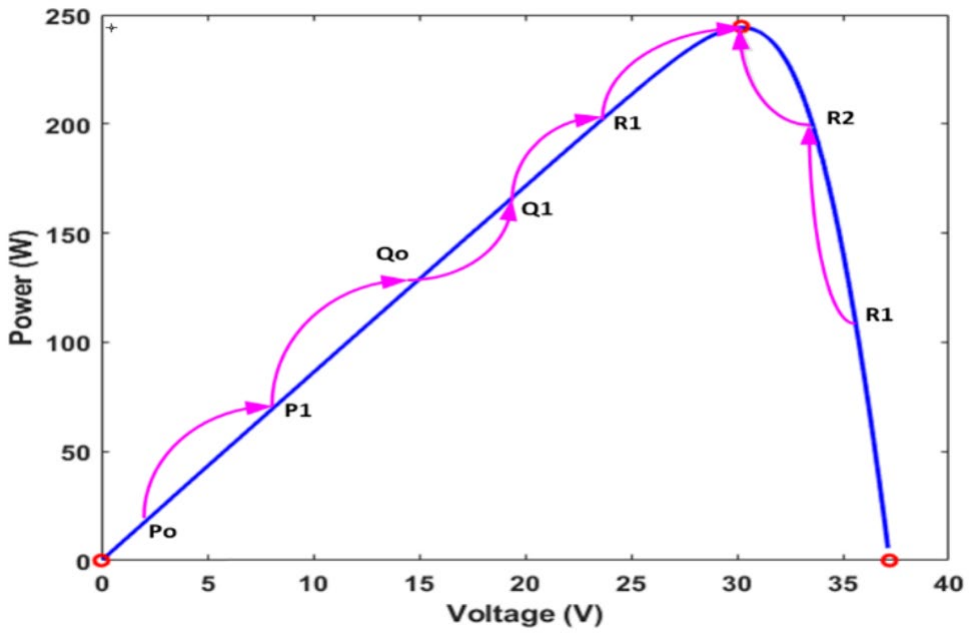
Power point tracking of solar PV by using CSA.

The current solution is ranked at each iteration until the stopping condition is met. When you use Levy flight search, you get a new solution, which can be written as the following Eq. (1). Where K values are 1, 2, and 3. The total number of samples is denoted by n. The following variable is step size, which is represented by the fitness function, which depends on the photovoltaic voltage, and shows how much power can be made ^49^. At first, samples are made and put on the solar panels that will later power us. This sets the first fitness value. The voltage value that produces the most power determines the best sample. Later, new voltage samples are made using the Levy flight search method, which is shown in the following Fig. 3.

CSO tracking of GMPP under steady and dynamic conditions is clearly shown in Fig. 3. The relationship between voltage and current in Fig. 3 shows the relationship between voltage and current without the partial shading effect of solar PV.Several samples are distributed over the PV curve, which is called the number of samples. When the number of samples isincreased the accuracy of MPP tracking is improved. When the number of samples isincreased, the search efficiency improves, but the convergence speed slows ^50^. As a result, four samples, such as P, Q, and R, are considered; additionally, the upper subscript indicates that iterations ^51^. As a result, it is the best solution for this iteration. So, these two samples are forced to move toward the best solution point. Because of the Levy flight mechanism, CS has a larger step size to achieve the best solution. Furthermore, the step size varies with each iteration, as shown in Fig. 3. It is clear that Qo is the best solution for the first iteration results, and P_o_ and R_o_ are resigning in favor of Q_o_^52^.

On the other hand, the next iteration R_1_ achieves the best solution because true MPP is very close to R_1_. To reach the global MPP of the solar PV system, the CS algorithm follows the steps outlined above. For the first iteration results, it is clear that Q_o_ is the best solution, and P_o_ and R_o_ are resigning in favor of Qo; however, the net iteration R_1_ achieves the best solution because of true MPP. Before starting a new iteration, a validation is done to see if the samples have reached convergence. Suppose samples are converged to an MPP result; there is no further iteration ^53^. To estimate the power, all of the samples' power quantities are measured. The samples with the highest power are referred to as the best samples ^54–60^. As a result, the remaining samples move in the direction of maximum power extraction from the solar PV systems. The step size is estimated by using the following Eq. (2).

2 $${{\text{V}}}_{{\text{k}}}^{{\text{s}}+1}={{\text{V}}}_{{\text{k}}}^{{\text{s}}}+\mathrm{\alpha }\oplus {\text{Levy}}(\uplambda )$$

3 $$\mathrm{\alpha }={\mathrm{\alpha }}_{0}({{\text{V}}}_{{\text{best}}}-{{\text{V}}}_{{\text{i}}})$$

The above Eq. (2) is used to find new solutions when it refers to the integral gamma function. If a sample gives less power than expected, it is ostensibly thrown out and a new one is made ^61^. These steps are repeated until the samples reach the MPP, which is the best place for them. When samples are found in the MPP results, the distance between them is zero, so all samples have the same value ^62^. This occurs only under a steady-state condition known as the "constant climate condition. But, practically, it's not possible. In this case, samples are created with varying power ranges for various voltages. At this moment, samples are once again disseminated over the power versus voltage curve. The process will be repeated until the weather settles down, which will cause ripples in the amount of power that is put out ^63^. Also, it is necessary to study the limitations of the cuckoo search MPPT controller.

The cuckoo search optimization technique uses search steps with a cluster of long jumps and multiple short steps to get the best solution ^64^. This process reduces the efficiency of the cuckoo search algorithm. Because of the long jump, the path is diverted to reach the true maximum power point, such as the global power point. As per the algorithm, there are immediately smaller steps, which cause very low voltage changes in the PV module. During long-step processes, the change in the duty cycle is very large, and the second-step process change in the duty cycle is very small ^65^. Furthermore, the path these actions suggest may lead to further investigation of the region close by. As a result, numerous iterations are needed to get to the GMPP, and in the meantime, the load will experience poor power oscillation. The cuckoo search algorithm locates nests by using random numbers ^66^. On fewer occasions, the location of nests is not well distributed in these areas. It results in repeated iterations, which causes the local solution to trump the global solution ^67^.

4 $${\upsigma }_{{\text{u}}}={\left(\frac{\Gamma (1+\upbeta )\times {\text{sin}}(\prod \times\upbeta /2}{\Gamma \left(\frac{1+\upbeta }{2}\right)\times\upbeta \times {\left(2\right)}^{\left(\frac{\upbeta -1}{2}\right)}}\right)}^{\frac{1}{\upbeta }}$$

## Design and modelling of Boost DC-DC converters

### Design and analysis of traditional boost converter

**Figure 4:**
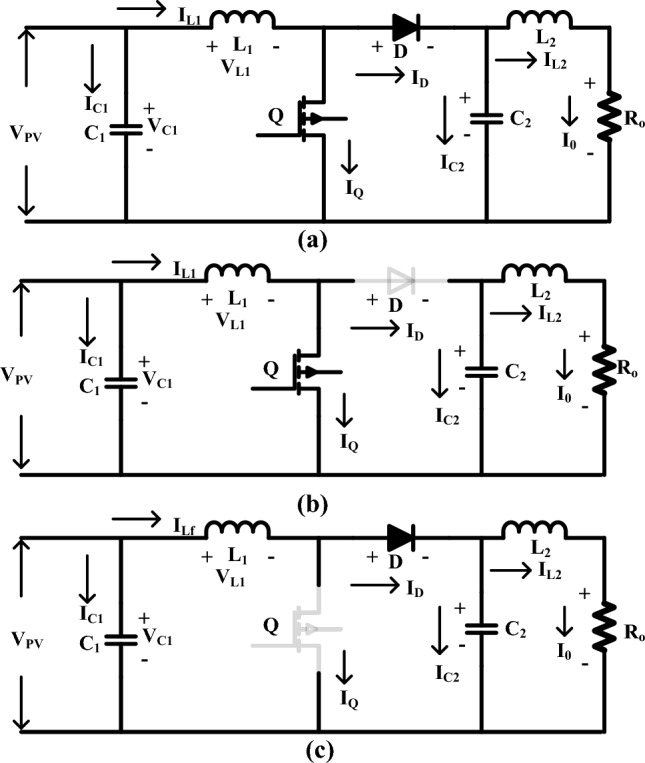
(**a**) Utilized converter structure, (**b**) Switching condition, and (**c**) OFF condition.

**Figure 5:**
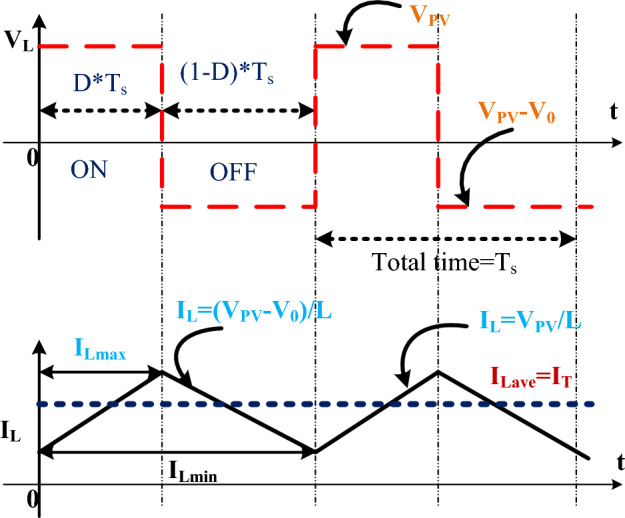
Boost converter duty cycle and inductor charging currents.

As shown in Fig. 4a, it is a common type of boost converter because it is as stable as others. It works in two modes, such as conducting, as shown in Fig. 4b, and blocking, as shown in Fig. 4c. In the first mode, the switch is on, which means it's forward biased, so the diode is operating in reverse bias condition at a time interval of dTs. Results: There is no supply transfer from source to load. During the DTS time, the switch is open, which makes the diode biased in the forward direction and moves power from the source to the load ^68–70^. The duty cycle operations are shown in Fig. 5.

Equation estimates the voltage balance equation of the input inductor. The current flowing through the inductor is obtained by using Eq. (5). Solving Eq. (5) and Eq. (6), which provide the output voltage and current of the boost converter as shown in Eqs. 7 and 8. Where RT refers to the resistance of the PV terminal. It is assumed that both resistances control the duty cycle. The design specification and corresponding formula are used to estimate the inductance and capacitance of conventional boost converters.

5 $${{\text{V}}}_{{\text{t}}}\times {{\text{Dt}}}_{{\text{s}}}+({{\text{V}}}_{{\text{t}}}-{{\text{V}}}_{0})\times (1-{\text{D}}){{\text{t}}}_{{\text{s}}}=0$$

6 $$-{{\text{I}}}_{0}{{\text{dt}}}_{{\text{s}}}+({{\text{I}}}_{{\text{Q}}}-{{\text{I}}}_{0})\times (1-{\text{d}}){{\text{t}}}_{{\text{s}}}=0$$

7 $${{\text{V}}}_{0}=({{\text{V}}}_{{\text{T}}}/(1-{\text{d}})$$

8 $${{\text{I}}}_{0}={{\text{I}}}_{{\text{T}}}(1-{\text{d}})$$

9 $$ {\text{V}}_{0}  = {\raise0.7ex\hbox{${{\text{V}}_{{\text{T}}} }$} \!\mathord{\left/ {\vphantom {{{\text{V}}_{{\text{T}}} } {(1 - {\text{d}})}}}\right.\kern-\nulldelimiterspace} \!\lower0.7ex\hbox{${(1 - {\text{d}})}$}}\& ;,{\text{I}}_{0}  = {\text{i}}_{{\text{T}}} \left( {1 - {\text{d}}} \right) $$

10 $$\frac{{{\text{V}}}_{0}}{{{\text{i}}}_{0}}=\frac{{{\text{V}}}_{{\text{T}}}}{{{\text{i}}}_{{\text{T}}}}\left(\frac{1}{{(1-{\text{d}})}^{2}}\right); ( \frac{{{\text{V}}}_{0}}{{{\text{i}}}_{0}}={{\text{R}}}_{0}, \frac{{{\text{V}}}_{{\text{T}}}}{{{\text{i}}}_{{\text{T}}}}={{\text{R}}}_{{\text{T}}})$$

11 $${{\text{R}}}_{{\text{T}}}={{\text{R}}}_{0}*\left({(1-{\text{d}})}^{2}\right)$$

### Design and analysis of switched switching boost converter

**Figure 6:**
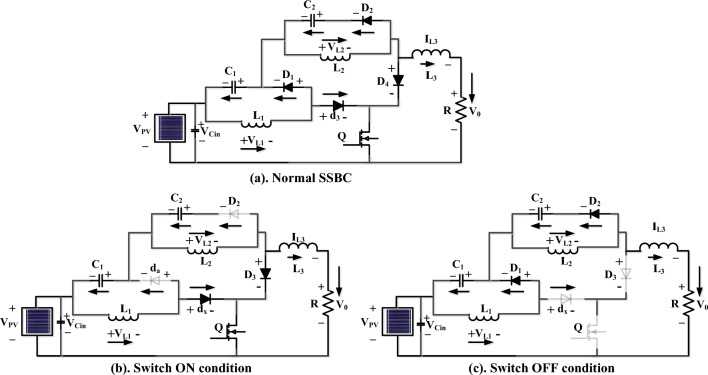
(**a**) Proposed SSBC, (**b**) SSBC conduction mode of operations, (**c**) Stackable switch boost converter (SSBC).

SSBC is important because it has a high voltage conversion ratio and makes very little oscillation in both static and moving conditions ^71^. Also, you don't need any magnetic parts to make the voltage signature better. Result: The cost of the converter is reduced marginally, as is the size of the converter. The proposed converter has two capacitors, four diodes, an inductor, and a switch. When the switch is turned on, the capacitor C_1_ charges, as shown in Fig. 6a. Whenever the switch is closed, current flows to the capacitor C_1_ through diodes D_3_ and D_1_, as shown in Fig. 6b. Also, inductor L_1_ stored electrical energy in its magnetic field. These stored electrical energies are transferred to the load when diode D_3_ is in a biased condition. When diode D_4_ is in the "reverse biased" state, the energy stored in capacitor C_2_ and inductor L_2_ is sent to the load ^72^. This can be expressed mathematically by following Eqs. (12) to (15). When the switch is turned off, as shown in Fig. 6c. The diode D_1_ is forward-biased, and the capacitor C_1_ begins to charge. Results, inductor current, and voltage across the capacitor are estimated by the following relations: (16) to (19).

12 $${{\text{L}}}_{1}\left(\frac{{{\text{di}}}_{1}}{{\text{dt}}}\right)={{\text{V}}}_{{\text{spv}}}$$

13 $${{\text{C}}}_{{\text{a}}}\left(\frac{{{\text{dV}}}_{{\text{c}}1}}{{\text{dt}}}\right)=-{{\text{I}}}_{{\text{L}}1}-\left(\frac{{{\text{V}}}_{{\text{o}}}}{{\text{R}}}\right)$$

14 $${{\text{L}}}_{2}\left(\frac{{{\text{di}}}_{{\text{b}}}}{{\text{dt}}}\right)={{\text{V}}}_{{\text{spv}}}+{{\text{V}}}_{{\text{c}}1}$$

15 $${{\text{C}}}_{2}\left(\frac{{{\text{dV}}}_{{\text{c}}2}}{{\text{dt}}}\right)=-\frac{{{\text{V}}}_{{\text{o}}}}{{\text{R}}}$$

16 $${{\text{L}}}_{1}\left(\frac{{{\text{d}}}_{{\text{i}}1}}{{\text{dt}}}\right)=-{{\text{V}}}_{{\text{c}}1}$$

17 $${{\text{C}}}_{1}\left(\frac{{{\text{dV}}}_{{\text{c}}1}}{{\text{dt}}}\right)=-{\text{IL}}1-\left(\frac{{{\text{V}}}_{{\text{o}}}}{{\text{R}}}\right)$$

18 $${\text{L}}2\left(\frac{{{\text{dV}}}_{{\text{c}}2}}{{\text{dt}}}\right)=-{{\text{V}}}_{{\text{c}}2}$$

19 $${\text{C}}2\left(\frac{{{\text{dV}}}_{{\text{c}}2}}{{\text{dt}}}\right)={\text{IL}}2-\left(\frac{{{\text{V}}}_{{\text{o}}}}{{\text{R}}}\right)$$

From inductor L_1_, the stored energy is transferred to the load with the slope of V_c1_/L_1_. Likewise, L_2_ releases the energy with a slope of V_c2_/L_2_. The voltage across the SCB's load resistance is the sum of the energy voltages across the capacitors Vc1 and Vc2, as well as the solar PV voltage. Figure 6 shows on a graph how the current flows through the inductor and how much voltage is across the capacitor as the switches are turned on and off. It showed. Whoever turns on the switch immediately charges the inductors L1 and L2, whereas it releases stored energy through capacitor C1 and inductor L2. Using the voltage and current harmonics, the slope of the energy storage element can be estimated. The voltage across each element is estimated by the following equations: (20) to (23).

20 $${\text{L}}1\left(\frac{{{\text{di}}}_{1}}{{\text{dt}}}\right)={{\text{dV}}}_{{\text{spv}}}-(1-{\text{d}}){{\text{V}}}_{{\text{c}}1}$$

21 $${{\text{C}}}_{1}\left(\frac{{{\text{dV}}}_{{\text{c}}1}}{{\text{dt}}}\right)=-{{\text{d}}}_{{\text{iL}}2}+(1-{\text{d}}){{\text{I}}}_{{\text{L}}1}-\left(\frac{{{\text{V}}}_{{\text{o}}}}{{\text{R}}}\right)$$

22 $${{\text{L}}}_{2}\left(\frac{{{\text{d}}}_{{\text{i}}2}}{{\text{dt}}}\right)={\text{d}}({{\text{V}}}_{{\text{spv}}}+{{\text{V}}}_{{\text{c}}1})-(1-{\text{d}}){{\text{V}}}_{{\text{c}}2}$$

23 $${{\text{C}}}_{2}\left(\frac{{{\text{dV}}}_{{\text{c}}2}}{{\text{dt}}}\right)=(1-{\text{d}}){{\text{i}}}_{{\text{L}}2}-\left(\frac{{{\text{V}}}_{{\text{o}}}}{{\text{R}}}\right)$$

## Parameter selection of optimization controllers

The PSO algorithm finds the best solution in two ways: first, the particle itself finds the best solution by moving towards it, and second, the entire population finds the best solution, respectively. The PSO optimization is based on the following two Eqs. (24) and (25).

24 $${\upeta }_{{\text{i}}}({\text{x}}+1)={\text{w}}{\upeta }_{{\text{i}}}({\text{x}})+{{\text{c}}}_{1}{{\text{r}}}_{1}({{\text{Pbest}}}_{{\text{i}}}-{{\text{q}}}_{{\text{i}}}({\text{x}})+{{\text{c}}}_{2}{{\text{r}}}_{2}[{\text{Gbest}}-{{\text{q}}}_{{\text{i}}}({\text{x}})]$$

25 $$ {\text{qi}}\left( {{\text{s}} + {1}} \right) = {\text{qi}}\left( {\text{x}} \right) + \lambda {\text{i}}\left( {{\text{x}} + {1}} \right) $$

where qi is the position of the i^th^ particle. ${\eta }_{i}$ is the velocity of the i^th^ particle. k is the iteration number. w is the inertia weight. r1 and r2 are the random variables and it is uniformly distributed within [0,1]. c1 and c2 are the acceleration coefficients. Pbesti is used to store the best position of ith particle. Gbesti is the best position if all particles in the entire population.

From the Eq. 1 and 2 observed that the position of the particle and velocity have a direct correlation, respectively. The new position $qi(s+1)$ is the sum of the old position and the new velocity $\lambda i(x+1)$ it can be estimated by the Eq. (1).The moment of the particle is decided by the objective function here it is called fitness value evaluation. For each reference voltage, there is a corresponding power it is called a fitness function to decide the movement ^73–78^.The position of the particles is adjusted by the following Eqs. (26).

26 $${\text{f}}({{\text{q}}}_{{\text{i}}}+1)>f({{\text{P}}}_{{\text{best}}})$$

To implement the above process for MPPT

27 $${{\text{q}}}_{{\text{i}}}({\text{k}}+1)=[{{\text{V}}}_{{\text{sp}}1},{{\text{V}}}_{{\text{sp}}2}......{{\text{V}}}_{{\text{spn}}}]$$

where, Vsp1, Vsp2 …… Vspn is called a particular particle as mentioned in Eq. 27, several studies proved that only three number particlesprovidean accurate and fast solution. Therefore, here three particles are used such as Vsp1, Vsp2, and Vsp3, respectively. These sample voltages are taken from the P–V and I-V characteristics of solar PV and it covers the entire curve.

28 $${{\text{S}}}_{\text{pv}}({{\text{V}}}_{\text{spvi}}({\text{x}}))> \text{SP} ({{\text{V}}}_{\text{spvi}}(\text{x-1}))$$

From the above Eq. 28, the objective function is power generated by the PV array is larger than that of the previous power subsequently particle movement is initiated otherwise vice versa. The inertial weight is represented as w and it is 0.4 and the local learning coefficient and global learning coefficient are represented as c1 and c2 these values are equal to 1.2. These two parameters are used to find the best local and global maximum power point, respectively.At first, a voltage sample is initiated because it is the solution vector. Meanwhile, the estimated duty cycle at this moment is transmitted to the converter. Also, all the particles are moving towards their local best solution. However, Gbest is also present among these particle solutions. Hence, it offered the best fitness value compared to all other solutions. Subsequently, a new velocity has been estimated using perturbations to the voltage, resulting in a new voltage position. All particles progress toward the global optimal position during subsequent iterations. When particles reach the MPP result, they close at the Gbest position. Therefore, the local best solution's velocity and the Global best solution’s velocity become zero. Finally, MPP is reached in this situation, velocity becomes zero, and velocity position is unchanged ^79,80^.The flower pollination MPPT algorithm is developed based on the following rules. The local pollinators are biotic and self-pollinating. The potential for reproduction varies according to flower constancy. It correlates with similar flowers undergoing pollination. The second probability controls local and global pollination. The global pollutant sources are biological and cross-pollinated; few flowers fly a long distance and obey levy flights. To develop a mathematical model of flower pollination by using global pollination and flower consistency, which are expressed as the following equations:

29 $${{\text{K}}}_{{\text{i}}}^{{\text{t}}+1}={{\text{K}}}_{{\text{i}}}^{{\text{t}}}+\mathrm{\gamma L}(\uplambda )({\text{H}}-{{\text{K}}}_{{\text{i}}}^{{\text{t}}})$$

where ${{\text{K}}}_{{\text{i}}}^{{\text{t}}+1}$, is a solution vector at iteration t. The best solution forthe boost converter duty cycle is H. The step size can be controlled by the $\upgamma$. The best step size of the levy flight search is represented using ${\text{L}}(\uplambda )$. However, the incest may fly long distances and it can be expressed in Eqs. (30), and (31).

30 $${\text{L}}\approx \frac{\mathrm{\lambda \Gamma }(\uplambda ){\text{sin}}(\frac{\mathrm{\Pi \lambda }}{2})}{\Pi }\frac{1}{{{\text{Q}}}^{1+\uplambda }}({\text{Q}}>>{{\text{Q}}}_{0}>0)$$

$\Gamma (\lambda )$ called as gamma function.The local pollination can be expressed by the Eq. (31)

31 $${{\text{K}}}_{{\text{i}}}^{{\text{t}}+1}={{\text{K}}}_{{\text{i}}}^{{\text{t}}}+\in ({{\text{K}}}_{{\text{x}}}^{{\text{t}}}-{{\text{K}}}_{{\text{y}}}^{{\text{t}}})$$

where $\mathrm{{\text{K}}}_{{\text{x}}},{{\text{K}}}_{{\text{y}}}$ representing the similar spices pollen from the different flowers. The switching probability is a crucial factor that should be used effectively to switch between global and local solutions. Here the number of population size can be represented as 3. Moreover, the number of iterations taken is 10. The crucial factor probability of the switch value is assumed 0.3. The global search is performed by the probability of switch whereas the local search is performed by the proximity probability. The pollination value 0.3 represents 30% algorithm doesa local search and remining will do a global search respectively. The boundary of the solution vector lies between 0 and 1.

## Simulation results and analysis

**Figure 7:**
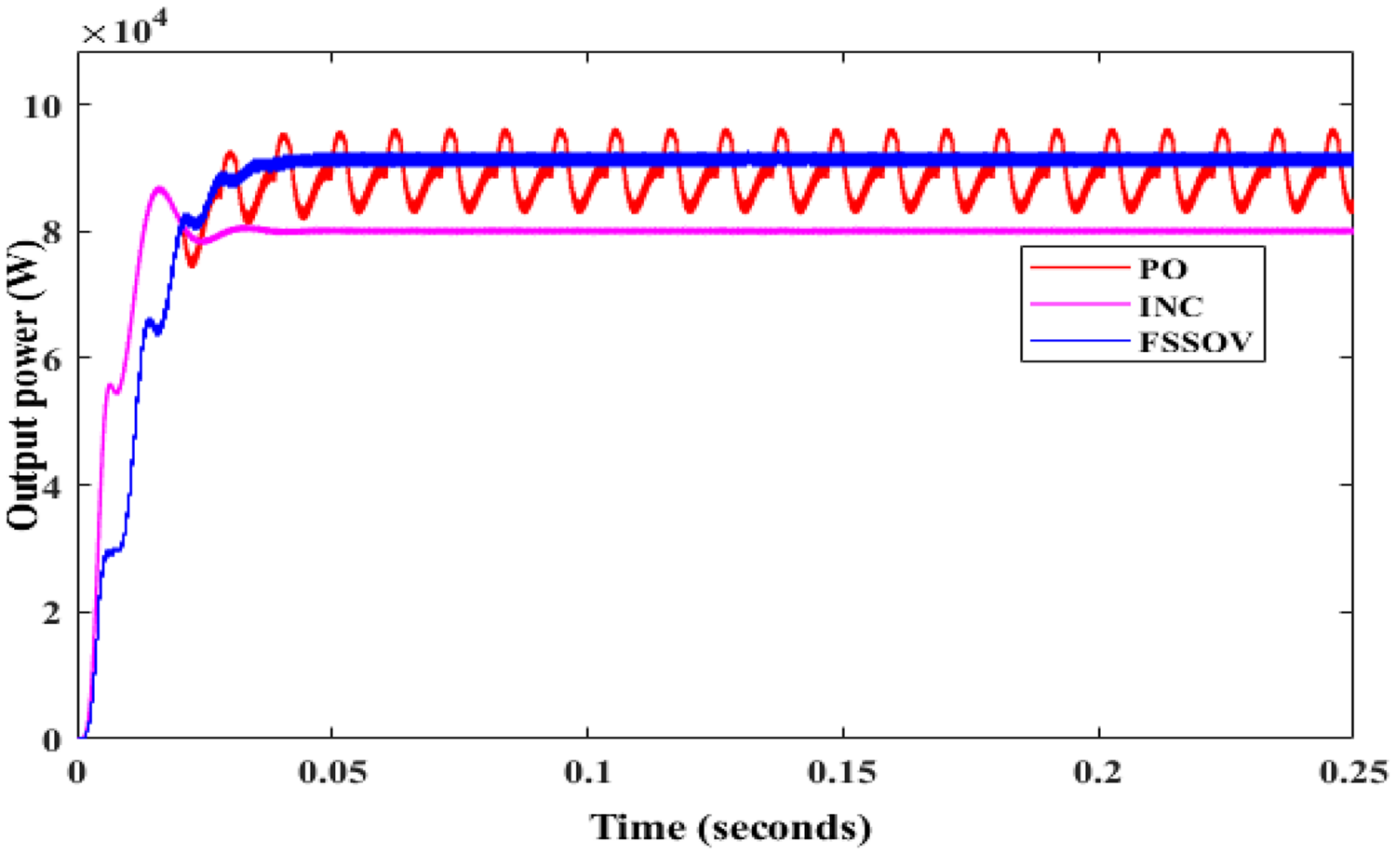
CBC along with CMPPT controllers output power.

**Figure 8:**
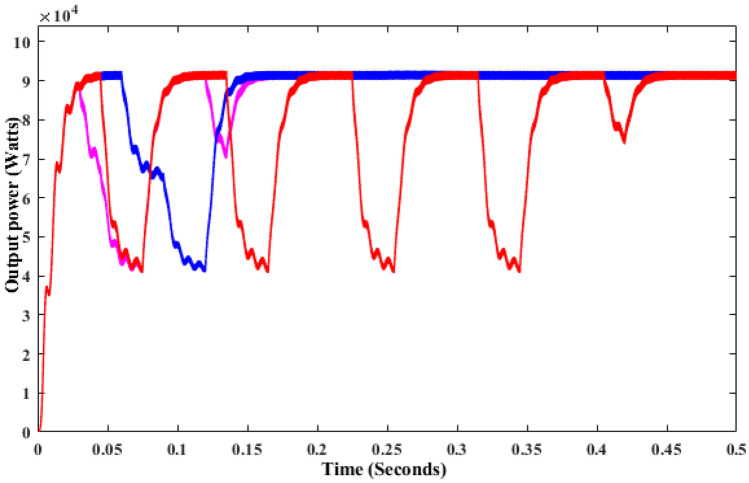
CBC with BMPPT controllers output power.

At first, the simulation study was carried out on a conventional boost converter along with the conventional MPPT controllers such as Perturb & Observe (P&O), Incremental Conductance (INC), VSS, and FSSOV at 1000 W/m^2^ for estimating converter efficiency. From the simulation study, it was found that the P&O algorithm produced huge oscillation whereas INC and FSSOV produced very less oscillations as shown in Fig. 7. The INC produced lesser power which is 8.1324*e^4^ W whereas FSOSOV produced 9.554*e^4^W and 9.261*e^4^W by INC Fig. 7. The time required to reach a stable value for FSSOV is 0.036 s 0.0356 s is required for INC. The simulation was also carried out with bio-inspired MPPTs. As shown in the Fig. 8, FPNA produced a large oscillation, it took 0.435 s to reach the stable value. While, CSO MMPT took 0.161 s to reach stable whereas 0.161 s is required for PSO to reach a stable value, respectively. The CSO tracks the high-power output from the solar PV compared to FPNA and PSO as shown in Table 1.

**Table 1:** Comparative analysis of CBC with MPPT’s.

S. no	MPPT	Vin	Vout	Pin	Pout	Efficiency
1	PO	286 V	417.2 V	9261W	8760W	94%
2	INC	322.2 V	396.6 V	8132W	7913W	97%
3	FSSOV	266.3 V	420.5 V	9554W	8919W	93%
4	CSO	239.5 V	391 V	8606W	7843W	91%
5	PSO	239 V	392 V	8581W	7880W	91%
6	FPNA	219.3 V	371.8 V	790W	7143W	90%

**Figure 9:**
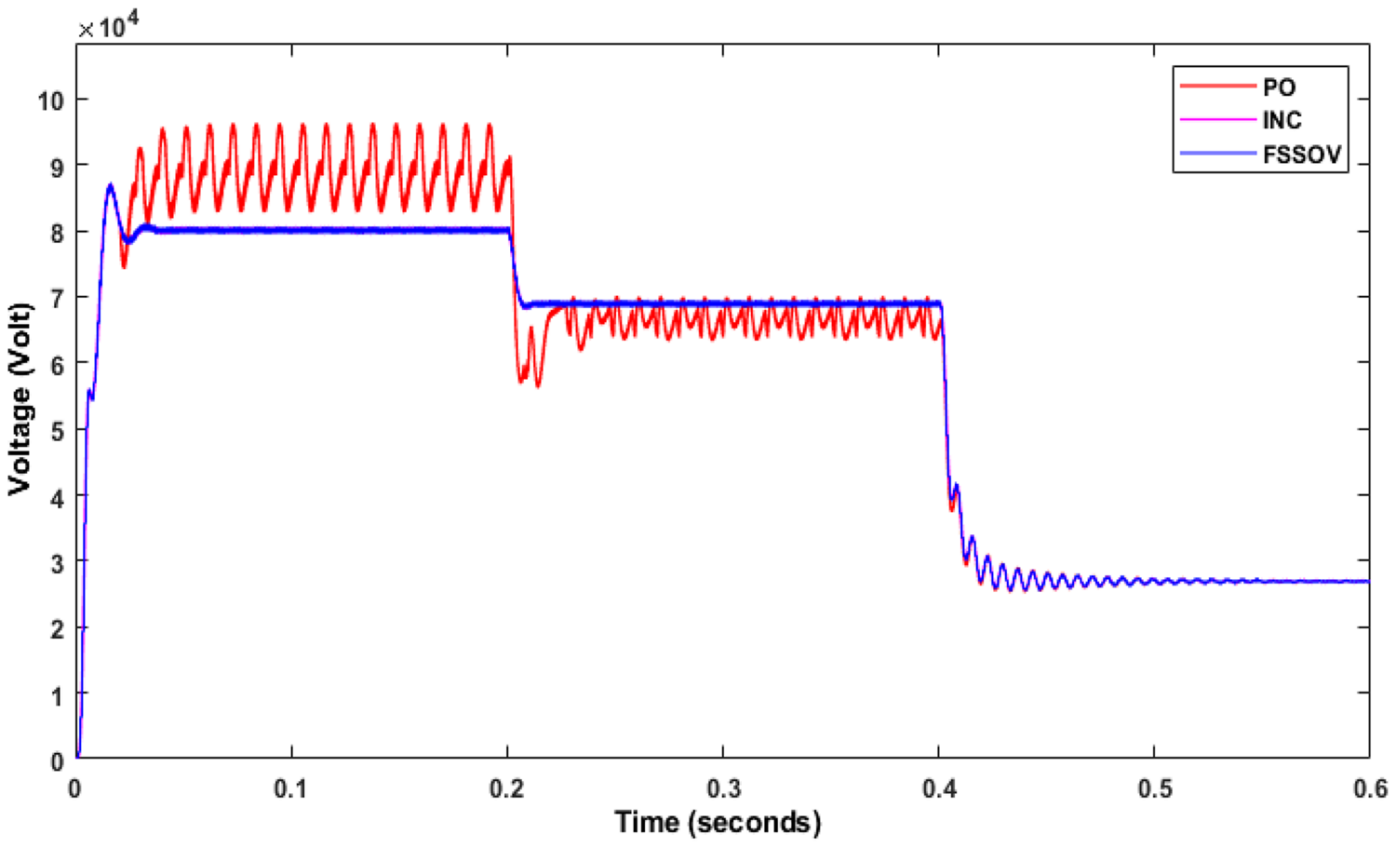
CBC with CMPPT controllers output voltage.

**Figure 10:**
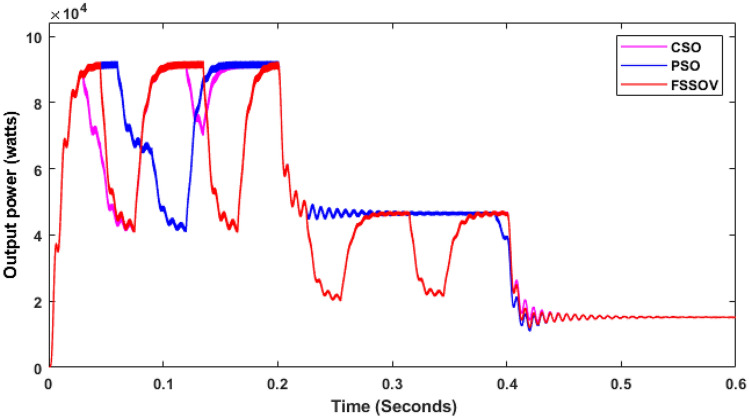
CBC with BMPPT under variable Irradiations.

In the environmental condition, the voltage varies continuously concerning time as shown in Fig. 9. As a result, the maximum power generated by the solar PV system varies. Hence, evaluation of the tracking efficiency of MPPT is essential, so the conventional boost converter with variable irradiation is simulated by using the MATLAB Simulink. The irradiation levels are 1000 W/m^2^ for 0 to 0.2 duty cycles, 700 W/m^2^ for 0.2 to 0.4 duty cycles, and 400 W/m^2^ for 0.4 to 0.6 duty cycles, respectively. Figure 10 depicts the relationship between voltage and current under variable irradiation. The PO-based MPPT oscillates more than the others. However, at 1000 W/m^2^, it produced483.9 V, which was reduced to 353 V at 700 W/m^2^ and 233.2 V at 200 W/m^2^, as shown in Table 2. Whereas 420.2 V is produced by the INC at 1000 W/m^2^, it produces 370.6 V at 700 W/m^2^ and 234.0 V at 200 W/m^2^. The FSSO is produced at 410 V at 100 W/m^2^, reduced to 367.0 V at 700 W/m^2^, and reached at 234 W at 200 W/m^2^ as shown in Fig. 10. The simulation revealed that P&O causes more oscillation at all levels of irradiation. FSSOV and INC, on the other hand, produce very few ripples in the output voltage as shown in Fig. 10.

**Table 2:** Comparative analysis of CBC under variable irradiations.

S. no	MPPT	Vin (V)			Vout (V)			Pin (W)			Pout (W)		
Irradiations		1000	700	200	1000	700	200	1000	700	200	1000	700	200
1	PO	325.5	313.7	193.0	438.3	353	233.2	8199	7049	2822	8049	6894	2739
2	INC	305.9	3.00	197.6	420.2	370.6	234.0	8274	7629	2578	8021	6990	2702
3	FSSOV	280.5	307	195.9	410.0	367.0	234.0	8256	6943	2579	8039	6909	2739
4	CSO	269.2	197.1	119.2	301.5	305.6	174.0	6235	4877	1751	9208	4608	1514
5	PSO	269.8	191.7	119.9	301.7	305.6	172.4	6023	4840	1753	9180	4677	1495
6	FPNA	203.5	194.4	117.5	336.2	306.5	179.7	7816	4877	1764	7196	495	1495

It is observed that the CSO-based MPPTs produce 9208 W at 1000 W/m^2^, which is reduced to 4608 W at 700 W/m^2^ and 154 W at 200 W/m^2^. The comparison between the effectiveness of CSO and PSO is very low. However, FSSOV performance is very poor under variable irradiation conditions. All MPPTs take time to track the maximum power during the first duty cycle. In the second duty cycle, PSO and CSO MPPTs track the maximum possible power faster than each other. Also, the tracking speed of CSO is superior to that of PSO under low irradiation.

**Figure 11:**
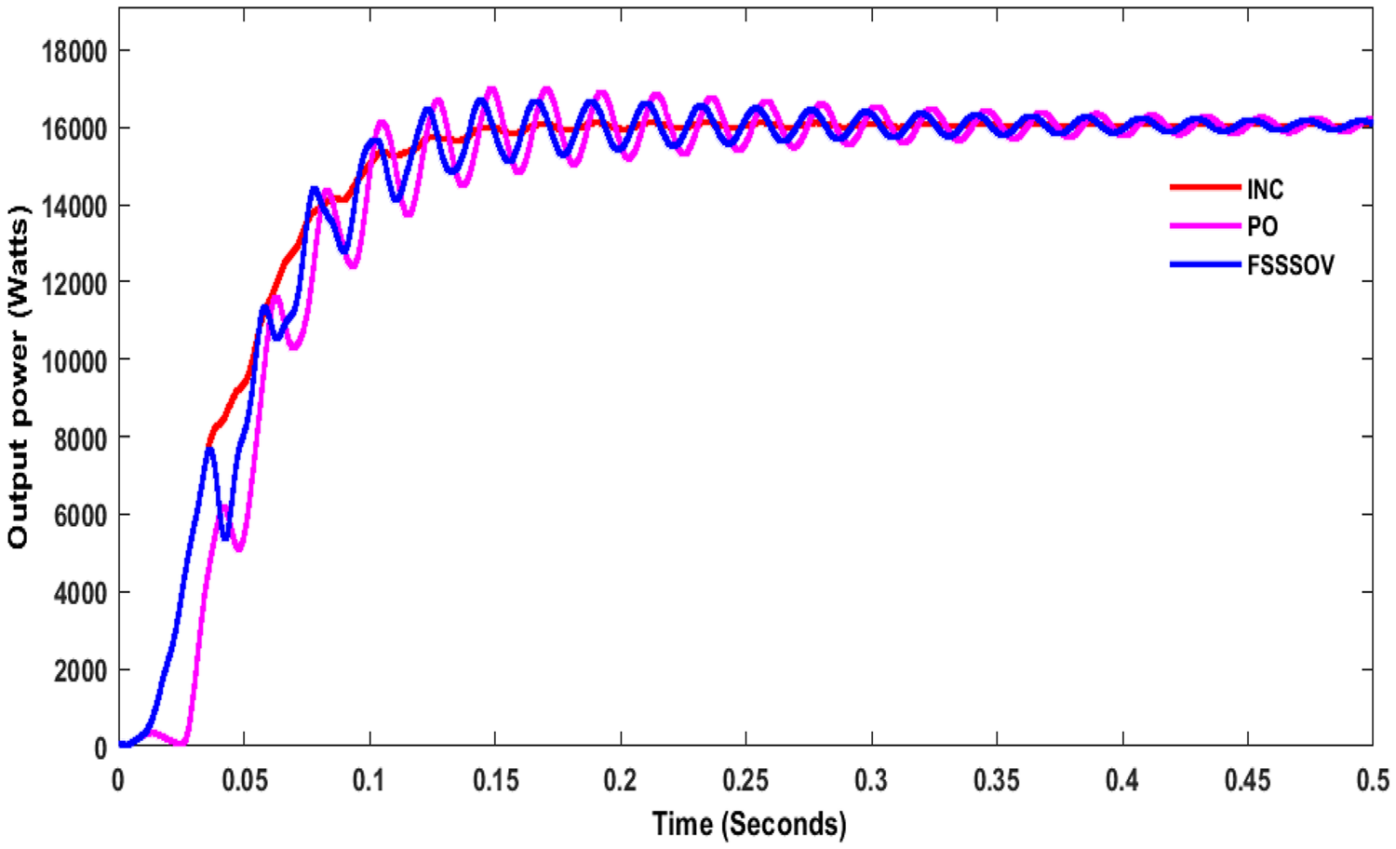
Output power of SSBC with CMPPT at constant irradiations.

**Figure 12:**
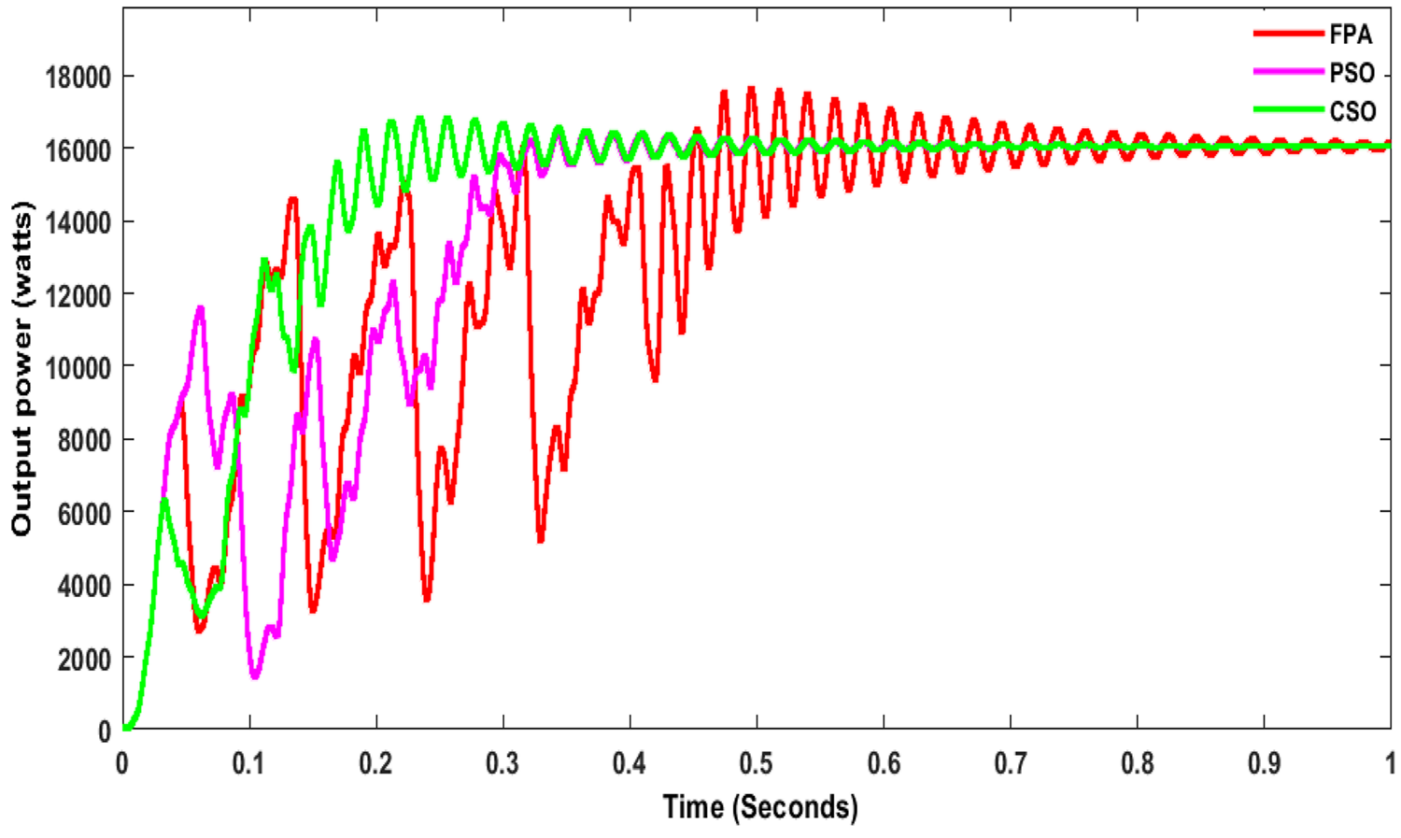
Output Power of SSBC with BMPPT at constant irradiations.

From the simulation, it was observed that P&O with SSBC produces huge oscillations, and it took 1 s to reach the stable voltage as shown in Fig. 11. The energy conversion range is 1.530 W with P&O as shown in Fig. 12. Whereas INC with SSBC is produced at 1450 W and takes 0.6 s to reach a stable value, FSSOV is produced at 1504 W and takes 0.85 s to reach a stable value. It is assumed that INC and SSBC provide the best performance. The SSBC with FPA produces 13,060 W and produces heavy oscillation as shown in Fig. 12. The SSBC with PSO, produces 13700W with much less oscillation than the FPA. Furthermore, PSO has a maximum power of 16,430 at 0.364 s, whereas FPA has a maximum power of 17,690 at 0.495 s. Within 0.256 s, the CSO with SCB produces a maximum of 16,840 W. In comparison to others, it produces 14,560 watts of average power. In addition, it produced much less oscillation than others. The output power of SSBC is given in Table 3.

**Table 3:** Performance of SSBC with MPPTs.

S. no	MPPT	Vin (V)	Vout (V)	Pin (W)	Pout (W)	Efficiency in %
1	PO	314	790.1	15,300	1297	84.7
2	INC	297.4	748.2	14,500	1.218	84
3	FSSOV	309	780.5	15,040	12,270	81.5
4	CSO	315.4	856.4	15,830	14,860	93.8
5	PSO	297.7	826.9	14,940	13,700	91.7
6	FPNA	285.1	806.4	1428 0	13,060	91.4

**Figure 13:**
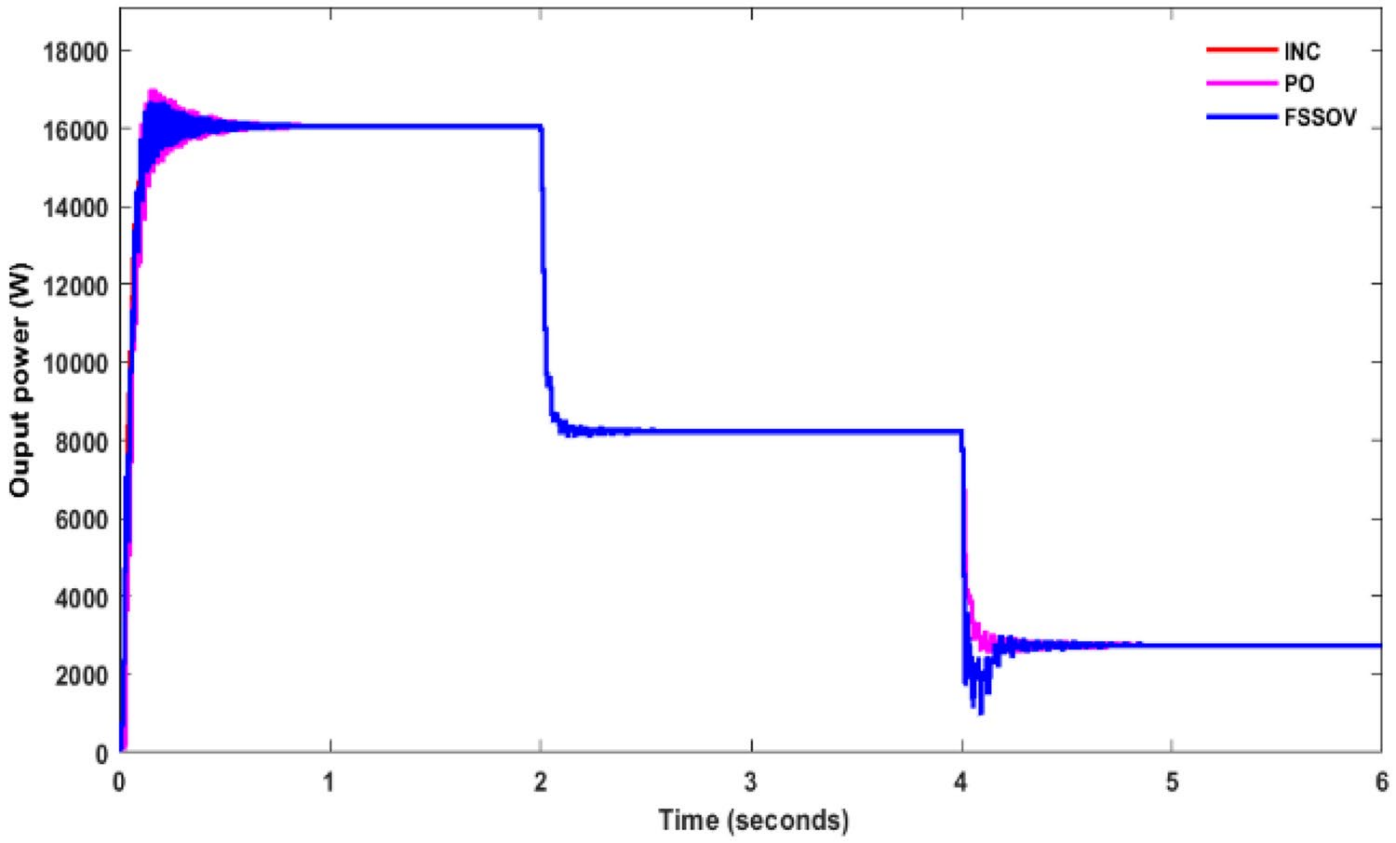
Output power of SSBC with CMPPT under variable irradiations.

**Figure 14:**
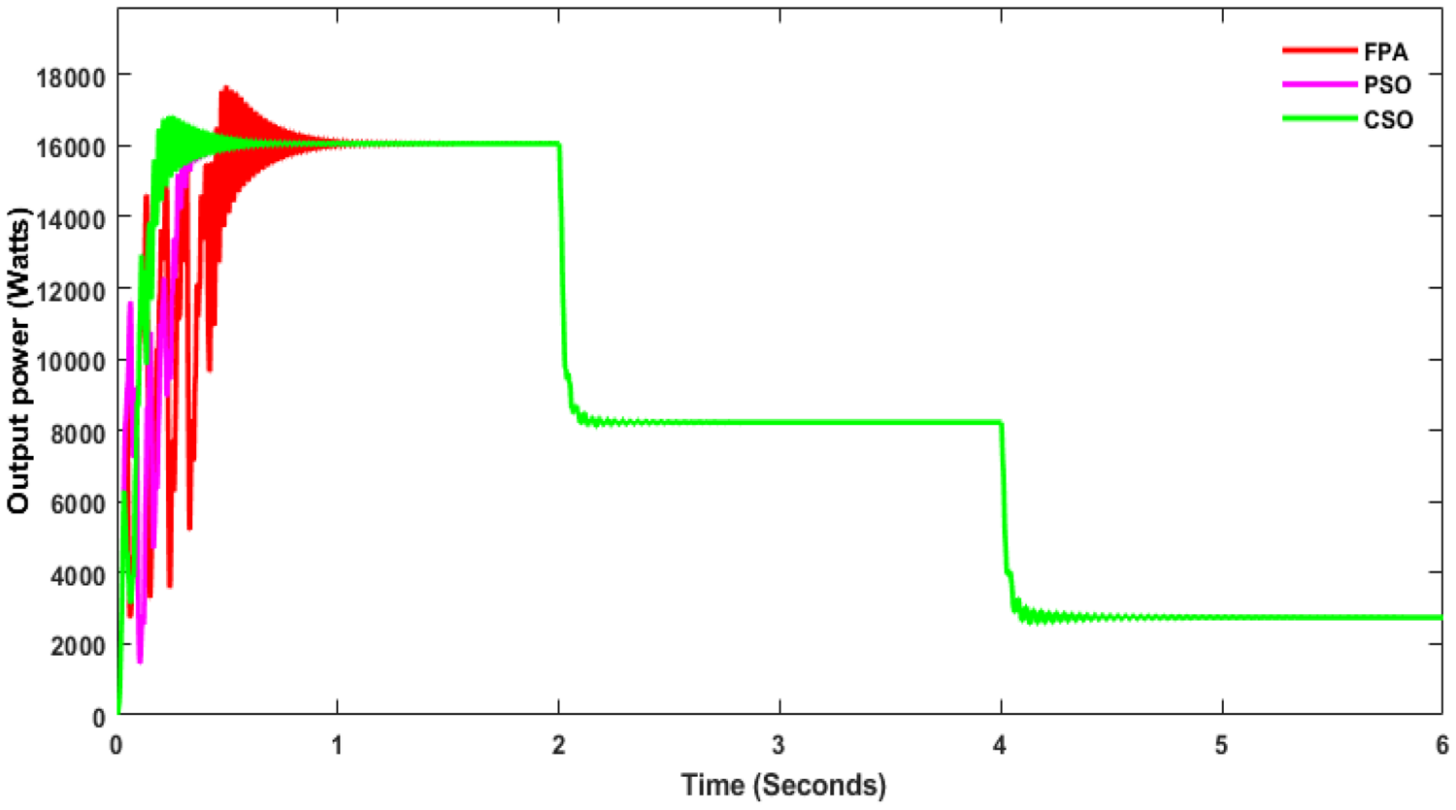
Output power of SSBC with TMPPT under variable irradiations.

As shown in Fig. 13, INC is 16,130 W at 1000 W/m^2^ whereas it is reduced to 8231 W at 700 W/m^2^ and finally, it is reached 2337 W at 400 w/m^2^ as shown in Fig. 13. The energy yield potential of the SSBC with CMPPT is almost equal for all the sampling irradiations but at low irradiation response time of P&O and INC MPPT is faster that of FSSOV. The conventional boost converter produced very little oscillation with the SSBC. As shown in Fig. 14, CSO with SSBC produces 17,690 W at 1000 W/m^2^ and it is reduced to 8759W at 700 W/m^2^, finally reaching 2739 at 400 W/m^2^. Also, it took 0.459 s to reach a stable power. Furthermore, it is worth noting that all BMPPT with SSBC produces nearly the same power. The voltage conversion ratio of SCB with BMPPT is comparable to that of SSBC with CMPPT. Furthermore, CSO with BMPPT produces significantly less oscillation than other methods. The efficiency of CSO with SSBC is higher than that of others as shown in Table 4.

**Table 4:** Performance of SSBC under dynamic irradiations.

S.no	MPPT	Pavg (W)	Pmax (W)	Time to reach maximum power in sec	Settling time in sec	Ripples in output voltage
1	FPA	9028	16,260	0.495	1.260	Moderate
2	CSO	9133	16,840	0.256	0.811	Low
3	PSO	9028	16,704	0.882	1.501	Moderate

**Figure 15:**
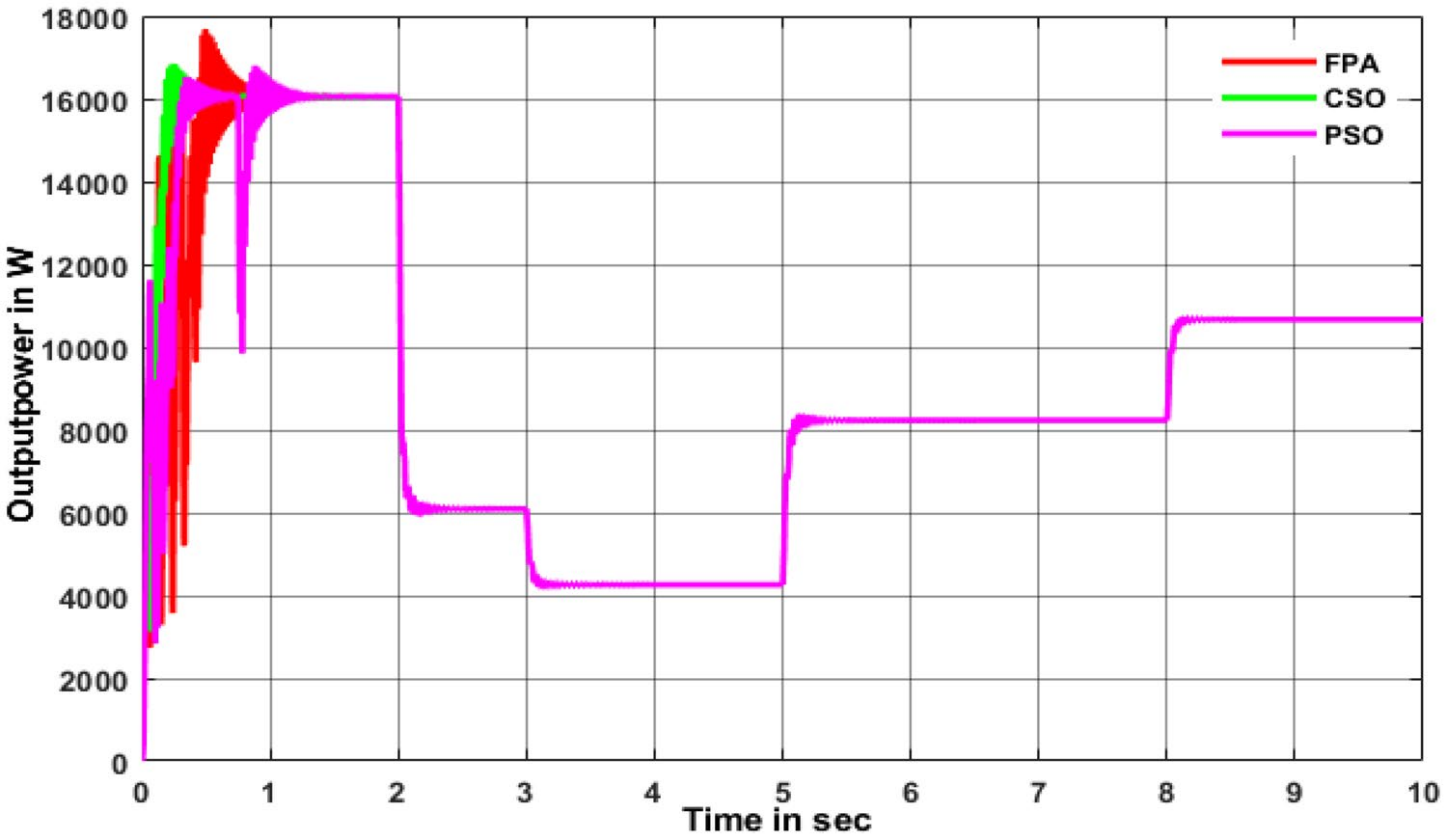
SSBC under dynamic irradiations.

The above critical studies found that the proposed converter with BMPPT’s efficiency is higher than that of others under steady and variable irradiations. Whereas, the performance of SSBC with BMPPT under dynamic irradiation is carried out in this section. It is essential, due to the change in irradiance on Earth, that the atmosphere is dynamic. Hence, dynamic irradiations such as 1000W/m^2^ for 0 to 2 s, 600W/m^2^ for 2 to 3 s, 500W/m^2^ for 3 to 5 s, 700W/m^2^ for 5 to 8 s, 800W/m^2^ for 8 to 10 s used for further analysis. The result shows that CSO MPPT with SCB produced higher power than others as shown in Fig. 15. In addition, the time to track the maximum power by CSO is 0.256 s which is lower than that of others. SSBC with CSO MPPT produces very low ripple power that of others. The FPA and PSO with SSBC produced moderate ripples on the output power. It is inferred that CSO is the best choice to extract the maximum power from solar PV under all the conditions, respectively.

## Conclusion

The research proposed an SSBC with CSO MPPT for the solar PV systems. It was observed that the P&O with CBC produced more ripples under constant and variable irradiations. Furthermore, FSSOV has produced a higher voltage than the other TMPPTs. It is evident from the results that the bio-inspired MPPT with CBC shows fewer ripples in the output power than the others. The efficiency of CSO with TBC is superior to that of PSO and FSSOV. The SSBC with TMMPT shows more efficiency of 93.8% when compared with other MPPTs. The results also reveal that the SSBC with BMPPT produced significantly higher output power than the TMPPT with SSBC. In addition, in dynamic conditions, CSO MPPT with SSBC took less time to reach a stable level than PSO and FFPA. Finally, when compared to the conventional boost converter, the proposed SSBC with CSO shows better results.

## Data Availability

The datasets used and/or analyzed during the current study are available from the corresponding author on reasonable request

## Abbreviations

SPV: Solar photovoltaic
MPPT: Maximum power point tracking
MPP: Maximum power point
CSO: Cuckoo search optimization
PO: Perturb and observe
INC: Incremental and conductance
FSSOV: Variable step size fractional incremental conductance
PSO: Particle swarm optimization
RER: Renewable energy resources
TBC: Traditional boost converter
Vt: Terminal voltage
VT: Total voltage
d: Duty cycle of conventional boost converter
IT: Terminal current
RT: Terminal resistance
SCB: Stackable switched capacitor
Vspv: Solar photovoltaic
L1: Input inductance
Ca: Input capacitor
IL1: Current flowing through inductor
Vo: Output voltage
L2: Smooth inductance
C2: Output capacitor
Vc1: Voltage across input capacitor C1
NMPPT: Bioinspired MPPT
FPNA: Flower pollination MPPT
CMPPT: Conventional MPPT
